# Lack of association of SNP rs4236601 near *CAV1* and *CAV2* with POAG in a Saudi cohort

**Published:** 2012-07-18

**Authors:** Khaled K. Abu-Amero, Altaf A. Kondkar, Ahmed Mousa, Essam A. Osman, Saleh A. Al-Obeidan

**Affiliations:** 1 Ophthalmic Genetics Laboratory, Department of Ophthalmology, College of Medicine, King Saud University, Riyadh, Saudi Arabia; 2 Department of Ophthalmology, College of Medicine, King Saud University, Riyadh, Saudi Arabia; 3 Glaucoma Unit, Department of Ophthalmology, College of Medicine, King Saud University, Riyadh, Saudi Arabia; 4 Department of Ophthalmology, College of Medicine, University of Florida, Jacksonville, FL

## Abstract

**Purpose:**

To determine the role of the recently discovered primary open angle glaucoma (POAG) single nucleotide polymorphism (SNP) rs4236601 near the caveolin-1 (*CAV1*) and *CAV2* among patients and controls from Saudi Arabia.

**Methods:**

A cohort of 220 POAG patients and 405 control subjects from Saudi Arabia were genotyped for a SNP (rs4236601;g.2891 G>A) in the chromosome 7q31 locus near *CAV1* and *CAV2* using a standard polymerase chain reaction (PCR) and sequencing method.

**Results:**

The minor allele frequency (MAF) of rs4236601 was 0.3 in controls and 0.31 in POAG patients. We detected no statistical difference when we compared the allele frequencies between POAG patients and control subjects (p=0.699). Similarly, we detected no statistical difference in the frequency of the three possible rs4236601 genotypes between patients and controls. The p-values were 0.928 and 0.683 for heterozygous genotype (G/A) and homozygous mutant genotype (A/A), respectively. We found no statistically significant difference among patients with any of the three possible genotypes and various clinical indices important for glaucoma. Among patients with homozygous (A/A), the mean IOP was higher (21.4) compared to patients with G/G wildtype (20.4) and to patients with G/A genotype (18.5). However, this apparent difference did not reach the statistical significance threshold (p=0.062).

**Conclusions:**

We were unable to detect this association in our POAG-patients from Saudi Arabia, suggesting that this risk factor may not have a strong effect in all populations. A founder effect may play a role in certain populations where the link was established.

## Introduction

Glaucomatous optic neuropathy is a chronic degenerative disease characterized by loss of retinal ganglion cells and their axons leading to progressive loss of vision [1,2]. With nearly 70 million people, being affected worldwide glaucoma is the second leading cause of blindness. This figure is estimated to reach 79.6 million by the year 2020 [3]. There exist wide variations in the prevalence of glaucoma among different ethnic groups and is significantly higher in blacks than in the white population [4]. Primary open angle glaucoma (POAG) is the most common form of glaucoma worldwide [5]. Although increased intraocular pressure (IOP) and vascular dysfunction are major risk factors for glaucoma, numerous other factors including age, race, gender, family history, oxidative stress, mitochondrial mutations, and specific gene mutations also contribute to the progression of the disease [6-10]. The major associated risk factors and the exact prevalence of POAG in Saudi Arabia are still largely unknown. However, in a recent study from King Abdul-Aziz University Hospital (KAUH), the glaucoma unit treats approximately 600 new glaucoma patients annually [11]. Of these patients, 19% were diagnosed with POAG, 40% with primary angle-closure glaucoma, 10% with pseudoexfoliation glaucoma and the remaining 31% with other miscellaneous types of glaucoma [11].

Several population and family-based studies support heredity of glaucoma to be a complex heterogeneous trait [12-15]. The heterogeneity can be attributed to several genes, the majority of which are yet unidentified, as well as the interactions of multiple genes with a variety of environmental factors. Genetic linkage analysis in large affected families has so far yielded 29 chromosomal loci linked to POAG. Of these, the Human Genome Organization (HUGO) genome nomenclature committee has designated 15 as GLC1A (GLC =Glaucoma; 1= primary open angle glaucoma; A = first linkage loci) to GLC1O and mutations in only four genes, which have been identified thus far. These include myocilin (*MYOC, GLC1A*), optineurin (*OPTN; GLC1E*), WD repeat domain 36 (*WDR36, GLC1G*), and neurotrophin-4 *(NTF4*, *GLC1O*) [16-20]. Recent studies have evaluated the role of genetic mutations and mitochondrial abnormalities in POAG among the Arab population [21,22]. Although these genes harbor POAG-associated mutations, they exhibit a high degree of allelic heterogeneity across populations and accounts for a small fraction of the cases. Furthermore, the variable penetrance and expressivity of the gene variants identified to-date do not shed much light on the underlying molecular mechanisms of the disease process.

Recently, Thorleifsson et al. [23] reported a genome wide association study that identified a strong association (p=5.0×10^−10^) of a common sequence variant (rs4236601) at chromosome 7q31 with glaucoma in the Icelandic population. The results were replicated in two different cohorts from UK, Sweden, and Australia (p=0.0015) of European descent and Hong Kong, Shantou, China (p=0.0021) of Asian origin. The risk variant is reported to be located near *CAV1* and *CAV2*. *CAV1* and *CAV2* are members of the caveolin gene family and their expression have been demonstrated in the trabecular meshwork [24] and retinal ganglion cells [25]. Furthermore, *CAV1* has been shown to be an important regulator of nitric oxide [26] and TGF-beta signaling [27] both of which have been implicated in the pathogenesis of POAG [27,28]. It would be of genetic epidemiological importance to evaluate whether the newly identified chromosome 7q31 risk variant predisposes the Middle-Eastern population of Saudi Arabia to POAG. The allelic and genotype frequency distribution of the rs4236601 variant at chromosome 7q31 and its association with POAG in the Saudi Arabian population have been examined in this study.

## Methods

### Patients and control subjects

We recruited 220 Saudi POAG patients (cases) who satisfied strict clinical criteria for POAG which includes the following: i) appearance of the disc or retinal nerve fiber layer e.g., thinning or notching of disc rim, progressive changes, nerve fiber layer defect; ii) the presence of characteristic abnormalities in visual field (e.g., arcuate scotoma, nasal step, paracentral scotoma, generalized depression) in the absence of other causes or explanation; iii) age greater than 40 years, and iv) open anterior chamber angles bilaterally on gonioscopy. Exclusion criteria included evidence of secondary glaucoma, e.g., pigmentary dispersion syndrome, pseudoexfoliation, history of steroid use or ocular trauma. Majority of POAG cases (213/220; 96.8%) had onset of glaucoma after 40 years of age (adult-onset POAG) and 7 cases (3.2%) had onset of glaucoma <40 years of age. Patients were recruited from the glaucoma clinic at KAUH, Riyadh, Saudi Arabia after signing an informed consent approved by the institutional review board (approval number # 08–657).

A second group (n=405) of healthy Saudi Arab controls free from glaucoma by examination were recruited. Inclusion criteria for these subjects were age >40 years, normal intraocular pressure (IOP), open angles on gonioscopy, and normal optic nerves on examination. The majority of our controls were >40 years of age (375/405; 92.6%) and 30 (7.4%) were <40 years of age.

The criterion for a “normal” IOP ranged from a minimum of 6 mmHg to a maximum of 21 mmHg. As the majority of eyes were put on anti-glaucoma medications, an eye was considered as with “uncontrolled” IOP if the IOP was >21 mmHg even with anti-glaucoma medications. Conversely, an eye was considered as with “controlled” IOP, if the IOP was within the above-mentioned normal range (6–21 mmHg) with or without anti-glaucoma medications.

### Sample collection and DNA preparation

Blood samples (5 ml) were collected in EDTA (EDTA) tubes from all participating individuals. The tubes were centrifuged at 5,500× g for 5 min. DNA was extracted from the buffy layer using the illustra blood genomicPrep Mini Spin kit (GE Healthcare, Buckinghamshire, UK) and stored at –20 °C in aliquots until further use.

### Single nucleotide polymorphism (SNP) genotyping

DNA samples were genotyped for rs4236601 by polymerase-chain reaction (PCR)-based direct automated sequencing [29]. PCR amplifications of the region encompassing the rs4236601 variant of the *CAV1* gene (NG_012051.1:g.2891G>A) were performed using the following primers (forward 5′-**TGT AAA ACG ACG GCC AGT** TAA AGG CCT CCC TGG AAA GT-3′ and reverse 5′-**CAG GAA ACA GCT ATG ACC** CAC CAC CAC ACC CTG CTA AT-3′; bold nucleotide are those of the M13 sequence). Successfully amplified fragments were sequenced in both directions using the M13 forward and reverse primers and the BigDye terminator v3.1 cycle sequencing kit (Applied Biosystems, Foster city, CA). Fragments were electrophoresed on the 3130*xl* Genetic Analyzer (Applied Biosystems) according to the manufacturer protocol. All the sequenced fragments were then analyzed using SeqScape software v2.6 (Applied Biosystems). Allele frequencies for rs4236601 variant were confirmed by repeating the sequencing in both the forward and reverse directions.

### Statistical analysis

Case to control sampling followed an estimated study power of 90%, α=0.05, critical T=1.96, standard moderate effect size 0.5 and CI=95% aiming at achieving both reliable and valid results based on concrete evidence.

Genotyping data were collected and stored in a spreadsheet using Microsoft Excel 2007^® ^(Redmond, WA). Data were linked and merged with our clinical registry glaucoma database at King Abdul-Aziz University Hospital using Microsoft Access 2007^®^. The new data set was imported, coded and analyzed using SPSS^®^ version 19.0 (IBM Inc., Chicago, IL), MedCalc^®^ 11.6 (MedCalc *S*oftware bvba, Mariakerke, Belgium) and StatsDirect^®^ statistical software, version 2.7.2 *(*StatsDirect Ltd., Cheshire, UK).

Visual acuity was converted from Snellen value to Logarithm of the Minimum Angle of Resolution (LogMAR) to suit the analysis purpose. Descriptive statistics including mean (SD) were calculated to describe continuous variables, while categorical variables were expressed as counted frequencies (number [%]). Comparison of means was done using one way ANOVA (ANOVA) test in case of continuous variables, Kruskal–Wallis H test was used instead of ANOVA when indicated, while χ^2^ test (Fisher exact test when indicated) was conducted to investigate the potential difference across groups in case of categorical data. A p-value less than 0.05 corresponding to 95% confidence interval was considered as indicator for statistical significance.

## Results

The total recruited sample size is 625 (mean (SD) age; 56.6 (12.5), range [11–93], male; 323 (51.7%), female; 302 (48.3%). Out of these 625 subjects, 220 subjects were recruited as cases (with confirmed diagnosis of primary open angle glaucoma) and the remaining 405 subjects served as controls. The case to control ratio was 1:1.84.

The mean (SD) age of cases was 61.4 (12.5), range [27–91] with males; 127 (57.7%) slightly exceeding females; 93 (42.3%). Among controls, the mean (SD) age was 54 (11.7), range [11–93], where females; 209 (51.6%) were slightly more than males; 196 (48.4%).

Among cases, the majority of subjects were of wildtype genotype (G/G); 110 (50%), followed by heterozygous (G/A); 83 (37.7%) and then homozygous mutant genotype (A/A); 27 (12.3%). Among controls, the majority of subjects were also of wildtype genotype (G/G); 207 (51.1%), followed by heterozygous (G/A) 153 (37.8%) and (A/A); 45 (11.1%).

On comparing genotypes between cases and controls, there were no statistically significant difference either for the heterozygous genotype (G/A); [OR: 1.02 (95% CI: 0.705–1.475), p=0.928] or the homozygous mutant genotype (A/A); [OR: 1.13 (95% CI: 0.636–1.975), p=0.638]. Additionally, there was no statistically significant association between having POAG and the type of allele [OR: 1.06 (95% CI: 0.813–1.366), p=0.699] (Table 1).

**Table 1:** Comparison of genotypes and allele distribution between cases and controls.

**Variable**	**Category**	**Cases No. (%)**	**Controls No. (%)**	**Total**	**OR**	**95% CI**	**p-value**
**Genotype**							
	G/G	110 (50)	207 (51.1)	317	-		
	G/A	83 (37.7)	153 (37.8)	236	1.02	[0.705–1.475]	0.928
	A/A	27 (12.3)	45 (11.1)	72	1.13	[0.636–1.975]	0.683
	Total	220	405	625			
**Allele**							
	G	303 (68.9)	567 (70)	870	-		
	A	137 (31.1)	243 (30)	380	1.06	[0.813–1.366]	0.699
	Total	440	810	1250			

Looking specifically at the group of cases, 208 (94.5%) out of the 220 were able to be linked with the clinical data at our clinical registry database, while 12 (5.5%) were either unable to link or having some missing data. After excluding those 12 cases, we conducted further analysis focusing on this group. Out of these 208 cases, 16 (7.7%) had a family history of glaucoma, 5 (2.4%) had consanguine parents, 99 (47.6%) had diabetes mellitus, 2 (1%) were smokers, 91 (43.8%) had hypertension, 1 (0.5%) had cardiac disease, and 3 (1.4%) had hypercholesterolemia. Other systemic diseases were also recognized among the cases group. We compared the three genotype groups in terms of different demographic and clinical characteristics at presentation, findings from these comparison are presented in details in Table 2 and Table 3.

**Table 2:** Comparing different genotypes of POAG patients in terms of their clinical characteristics.

**Variable**	**G/G Mean (SD)**	**G/A Mean (SD)**	**A/A Mean (SD)**	**Total Mean (SD)**	**p-value**
Age at presentation, years	63.7 (13.4)	61.5 (14.3)	63.1 (15.1)	62.9 (13.9)	0.373
Age at diagnosis, years (Onset/symptoms)	53.2 (14.1)	51.7 (14.5)	54.3 (13.5)	52.8 (14.1)	0.805
IOP, mmHg	20.4 (9.2)	18.5 (6.5)	21.4 (8.8)	19.9 (8.3)	0.062
Number of medications	2.1 (0.92)	2.2 (1.1)	1.7 (0.91)	2.1 (1)	0.058
LogMAR visual acuity	0.67 (0.91)	0.57 (0.78)	0.5 (0.75)	0.61 (0.84)	0.315
CDR (horizontal)	0.69 (0.22)	0.72 (0.26)	0.66 (0.21)	0.69 (0.24)	0.366

**Table 3:** Comparing different genotype categories of cases in terms of demographic and health status characteristics.

**Variable**	**Category**	**G/G n=107**	**G/A n=75**	**A/A n=26**	**Total n=208**	**p-value**
Sex	Male	60	43	15	118	0.980
	Female	47	32	11	90	
Family history of glaucoma	Yes	10	4	2	16	0.001
	No	97	71	24	192	
Consanguinity	Yes	2	2	1	5	0.826
	No	105	73	25	203	
Diabetes mellitus	Yes	54	34	11	99	0.116
	No	53	41	15	109	
Smoking	Yes	0	2	0	2	0.167
	No	107	73	26	206	
Hypertension	Yes	44	35	12	91	0.733
	No	63	40	14	117	
CAD	Yes	0	1	0	1	0.410
	No	107	74	26	207	
Hypercholesterolemia	Yes	2	0	1	3	0.318
	No	105	75	25	205	
Other systemic diseases	Asthma	1	1	0	2	0.511
	CHF	1	0	0	1	-
	Hepatitis B	1	1	0	2	-
	Hyperthyroid	1	0	1	2	-
	ITP	1	0	0	1	-
	Epileptic	0	1	0	1	-
	Vertiligo	0	1	0	1	-
	Aphakia	0	0	1	1	-
Aware of having glaucoma	Yes	38	27	8	73	0.883
	No	69	48	18	135	
Glaucoma Controlled	Yes	74	59	16	149	0.178
	No	33	16	10	59	

CHF=Chronic Heart Failure; ITP=idiopathic thrombocytopenic purpura.

Table 2 shows that there was no statistically significant difference among patients with the three genotypes in terms of the clinical indices at presentation. However, among patients with homozygous mutant genotype (A/A), the mean IOP was higher (21.4) compared to patients with G/G wildtype (20.4) and to patients with G/A genotype (18.5). However, this apparent difference did not reach the statistical significance threshold (p=0.062) although it went so critically close. Likewise, the mean number of used anti-glaucoma medication was also found to be slightly higher in the G/A group (2.2) than the G/G group (2.1) and the A/A group (1.7), but did not exceed the significance threshold showing p-value of 0.058 probably indicating a trend. These two important indicators may show statistically significant difference with a slightly larger sample size (Table 2).

On the other hand, when comparing the different demographic and health status indicators of glaucoma across the three genotype groups, having a family history of glaucoma was the only significant variable (p=0.001). The overall prevalence of cases with family history was 7.7% of all cases; meanwhile, the G/G group had the highest prevalence of this characteristic 9.3% than the other two groups (5.3% for the G/A and 7.7% for the A/A; Table 3).

Moreover, the overall awareness to being a glaucoma patient was 35.1% (almost one- third would know that they have glaucoma). According to genotypes, the G/G group showed an awareness prevalence of 35.6%, G/A that of 36% and A/A group of 30.8%. However, the difference was statistically insignificant (p=0.883). Similarly, the G/A group showed the best prevalence of controlled IOP (78.7%), which has much exceeded both G/G (69.2%) and A/A (61.5%) groups (Table 3).

## Discussion

In 2010, a genome-wide association study of subjects from Iceland mapped the first common genetic risk factor for POAG to a small region of the genome on chromosome 7q31 that contains the caveolin genes *CAV1* and *CAV2* . In this study, association between SNP rs4236601 and POAG was found among populations from Iceland, Australia, Hong Kong Chinese, and Shantou Chinese. In the same study, this association could not be established among Swedish and UK populations [23]. There have been two other published studies since then that have evaluated the association of this SNP with POAG. Both of the two studies have been reported among the Caucasians and have shown conflicting results. The first study was published in early 2011, where a US-cohort from Iowa consisting of 545 POAG patients and 297 control subjects were genotyped for SNP rs4236601. This study was unable to find a link between this SNP and POAG and suggested that this risk factor may not have a strong effect in all populations [30]. The second study was performed on 1000 POAG cases and 1183 controls collected as a part of the Gene Environment Association Studies (GENEVA) consortium [31]. This study reported a positive association of rs4236601 with POAG and more so, the effect was significant in women but not in men. To the best of our knowledge, no other study has investigated this association thus far.

We set out to determine the role of SNP rs4236601 as a risk factor for POAG among the Saudi Arabian population. Our results indicated that there was no difference in allele association (p=0.699) or genotype frequencies (p-values of 0.928 and 0.683 for G/A and A/A genotypes respectively) between cases and controls.

Since minor allele frequency (MAF) of SNP rs4236601 varies significantly between populations, association based on this SNP will be particularly sensitive to ethnic variability. The minor allele “A” frequency is 0.209 in the current default global population 1000 Genome phase 1 genotype data from 1094 worldwide individuals, released in the May, 2011 data set. Additionally, the HapMap database shows a great variability in MAF of SNP rs4236601 between populations. HapMap data shows that Asians have low minor allele frequencies (0–0.04), while Africans have high MAF (0.3–0.43). As for Hispanic and non-Hispanic Caucasians and an intermediate MAF rate was observed (0.22–0.29). In our Saudi cohort the MAF was 0.3 among controls group and this was slightly higher than the rate observed in US cohorts (0.27–0.28), Iceland (0.22), Sweden (0.27), UK (0.26), and Australia (0.26). Our MAF rate of 0.3 was much higher than the rates observed among Asians from Hong Kong (0.004) and Shantou Chinese (0.003) populations reported thus far in the two studies investigating the role of this SNP in POAG [30]. This indicates that there is high variability in the MAF rate among various populations, which suggest that this SNP will have a stronger link with POAG in some populations than others will. It is also possible that SNP rs4236601 is important to POAG in some populations than others due to a founder effect.

Then we proceeded with trying to link the genotyping data with various clinical indices. This is the first attempt in the literature trying to link the genotypes of SNP rs4236601 with various clinical indices important for glaucoma. We found no statistically significant difference among patients with various genotypes and this may be because of the relatively small sample size (n=208). We then compared different demographic and health status indicators of glaucoma across the three genotype groups, having a family history of glaucoma was the only significant variable (p=0.001).

Caveolin 1 and caveolin 2 are expressed in non-muscle cells while caveloin 3 is present in skeletal and a few muscle cells [32]. Immunohistochemistry showed caveolin expression in human retina, ciliary muscle, trabecular meshwork, and Schlemm’s canal. This makes it a plausible candidate gene for glaucoma. In an attempt to identify causative variant for POAG in the *CAV1/CAV2* genes, Wiggs et al. [31] had examined 50 Kb region flanking *CAV1/CAV2* and identified 14 additional SNPs and 2 haplotypes (of which rs4236601 was not a part of) with modest effects on POAG. Having said that, no glaucoma-causing mutations have been identified either in *CAV1* or *CAV2* to date. Failing to find a strong link between the recently discovered SNP rs4236601 by others and us [23,30] raises doubts about its importance in POAG pathogenesis, at least in some populations. However, as with many other complex disorders the interaction of genes with other confounding factors like age, gender (hormones), drugs affecting IOP may also have critical roles to play.

To conclude, it is fair to say that in our attempt to link this SNP with POAG among the Saudi population our study failed to establish a link. This leaves the true genetic cause of POAG among the Saudi population open for further investigation. Previously, we showed that the Saudi POAG patients lack mutation(s) in *MYOC*, *OPTN*, and *WDR36* [21]. Thus, a more comprehensive investigation using techniques such as genome wide association studies (GWAS) and recruitment of large number (at least 1,000 patients) of well clinically defined POAG patients potentially with age, gender, and ethnicity matched controls will be needed. Such a study has the potential to yield a better outcome of identifying gene(s) or SNPs associated with POAG in this population.
